# Ascending Cholangitis secondary to migrated embolization coil of gastroduodenal artery pseudo-aneurysm a case report

**DOI:** 10.1186/s12893-017-0227-9

**Published:** 2017-03-23

**Authors:** Haithem Zaafouri, Anis Hasnaoui, Sonia Essghaeir, Dhafer Haddad, Meriam Sabbah, Ahmed Bouhafa, Jamel Kharrat, Anis Ben Maamer

**Affiliations:** 1 Department of General Surgery Habib Thameur Hospital, Ali Ben Ayed Street’s 2037 Montfleury, Tunis, Tunisia; 2 Department of Radiology Habib Thameur Hospital, Tunis, Tunisia; 3 Department of Gastroenterology Habib Thameur Hospital, Tunis, Tunisia

**Keywords:** Gastroduodenal artery, Pseudo-aneurysm, Haemobilia, Embolization coil, Cholangitis

## Abstract

**Background:**

Gastroduodenalartery (GDA) pseudo-aneurysms are very rare. Their clinical importance lies in the eventuality of rupture, causing bleeding and ultimately exsanguination.

**Case presentation:**

We report the case of a man, with prior history of biliary surgery, presenting with haemobilia secondary to a rupture of GDA pseudo-aneurysm eroding the main bile duct. The patient was treated with coil embolization. This technique is considered to be safe. However, on the long term, some complications may occur. In our case, the patient presented with cholangitis subsequent to coil migration in the lower bile duct. This situation was managed using endoscopic retrograde cholangiopancreatography (ERCP) allowing coil extraction with favorable evolution.

**Conclusions:**

GDA pseudo-aneurysms are very rare. Bleeding, secondary to the rupture of these lesions, is a serious complication that could lead to death. Diagnosis and treatment of ruptured GDA pseudo-aneurysms rely on angiography. This method is considered to be safe. Cholangitis secondary to coil migration in the main bile duct is exceedingly rare,but remains an eventuality that physicians should be cognizant of.

## Background

Splanchnic artery aneurysms are rare entities. They are mainly cited in literature as case reports rendering their prevalence hard to determine [1]. Aneurysms of the gastroduodenal artery (GDA) are the least common [2]. Their clinical importance resides in the fact that they can be rapidly fatal if ruptured. The management of these conditions relies on endovascular embolization. This technique is considered to be safe. However, on the long term, seldom complications may occur. We report a case of upper gastrointestinal bleeding secondary to a ruptured pseudo-aneurysm of the gastroduodenal artery after choledochotomy, treated with endovascular embolization, and subsequent migration of a coil in the main bile duct, causing severe cholangitis.

## Case presentation

**Fig. 1:**
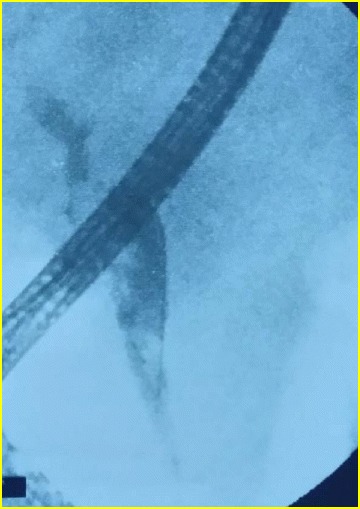
ERCP showing stones in the main bile duct

A 55-year-old man, with previous history of alcohol consumption, presented to our institution with a 6-day history of right upper quadrant pain, fever and progressive jaundice. Physical examination showed a temperature of 38 °C, a pulse rate of 98/min, a blood pressure of 10/7 cm Hg, scleral icterus and right upper quadrant pain with no rebound. Laboratory blood tests showed a leukocyte count of 12,300/ml, C-reactive protein of 215 mg/l, a conserved renal function. His liver function tests revealed a total bilirubin of 130 umol/l, an alanine transaminase (ALT) of 213 U/l, a gamma-glutamyltranspeptidase (γ-GT) of 686 U/l and an alkaline phosphatase (ALP) of 284 U/l. Amylase level and prothrombin time were normal. Abdominal ultrasonography foundcholelithiasis and a mild dilatation of both the extrahepatic and intrahepatic biliary tract. The obstacle was not identified. CT scan confirmed the presence of several stones impacted in the common bile duct, upstream biliary tree dilatation, and an enlarged pancreatic head with adjacent peripancreatic inflammation. We retained the diagnosis of acute pancreatitis with ascending cholangitis. The patient was admitted and antibiotics were then intravenously administered. Twenty-four hours later, he had an endoscopic retrograde cholangiopancreatography (ERCP) with sphincterotomy and stones extraction with favorable evolution (Fig. 1). The patient was discharged 3 days later.

A laparoscopic cholecystectomy was scheduled, after regression of peripancreatic inflammation, 3 months later. Due to dissection difficulties, we converted to a Kocher’s incision to complete the cholecystectomy. Transcystic cholangiography showed residual stones. We performed choledocotomy with two stones retrieval and T-tube choledochostomy. Immediate post-operative course was uneventful and T-tube cholangiography was normal. The patient was discharged in the 5^th^ post-operative day.

Two weeks later, the patient presented with hematemesis and melena. On examination, he had icterus, blood pressure of 12/8 cm Hg and a pulse of 85 beats per minute. On digital rectal examination, he had melena. There was no blood exteriorization from the T-tube. Blood tests showed a hemoglobin level at 96 g/l, a total bilirubin of 46 umol/l, an ALT of 225 U/l, a γ-GT of 516 U/l. He underwent upper gastrointestinal endoscopy showing mycotic esophagitis and erosive bulbitis with no active bleeding. Lateral duodenoscopy revealed no bleeding from the papilla. CT scan showed infiltration of sub-hepatic fat and magnetic resonance cholangiopancreatography was normal. During hospitalization, the patient was hemodynamically stable, without further drop in hemoglobin level. He was discharged with an appointment to our outpatient department.

**Fig. 2:**
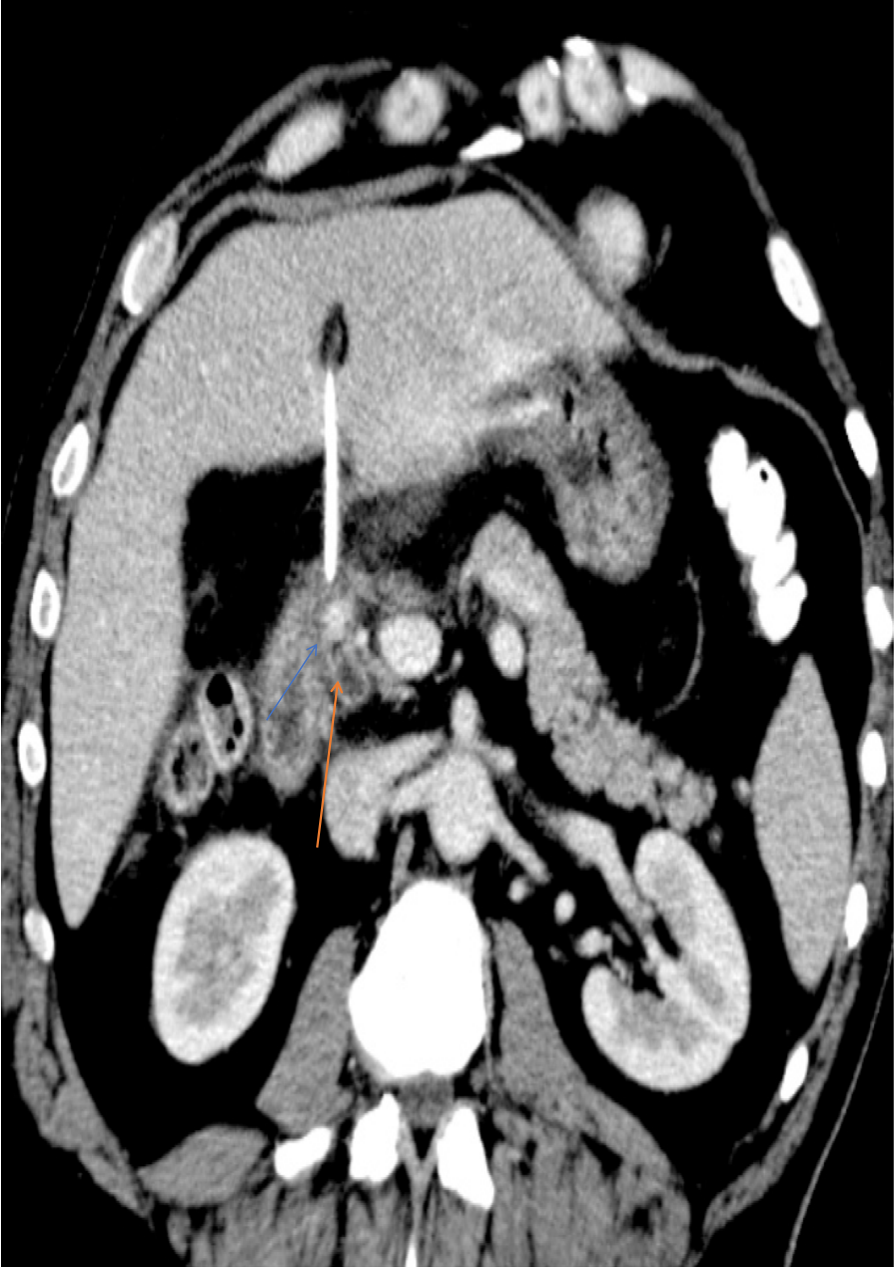
CT-angiography showing a pseudo-aneurysm of the gastroduodenal artery with probable erosion of the main bile duct

After 1 month of being discharged, the patient presented with recurrence of upper gastrointestinal bleeding and haemobilia with blood exteriorization from the T-tube. On examination, he was hemodynamically stable, he had scleral icterus and right upper quadrant pain. On digital rectal examination, he had melena. Hemoglobin level was at 85 g/l and his liver function tests revealed icteric cholestasis. A CT-angiography was performed showing a pseudo-aneurysm of the gastroduodenal arterywith probable erosion of the main bile duct (Fig. 2).

In the third day post admission,a massivebleeding and hemorrhagic shock occurred. Hemoglobin level was at 40 g/l. The patient was admitted to the intensive care department. After resuscitation and transfusion his condition was relatively stabilized, and he was addressed for urgent embolization. Coeliac arteriography confirmed the bleeding from the pseudo-aneurysm of the gastroduodenal artery. Embolization using three coils achieved successful hemostasis with preserved permeability of the gastroduodenal artery. The immediate post-embolization period passed without any complications. The patient was discharged 6 days later after T-tube removal. Then he was lost to follow-up.

**Fig. 3:**
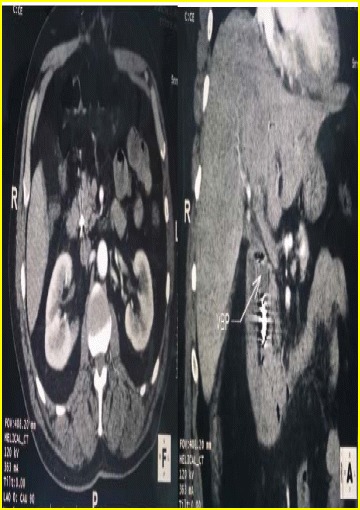
Axial (*Left picture*) and Frontal (*right picture*) CT-scan images, showing a hyper-dense obstacle in the bile duct

Twenty months later, the patient presented with Charcot’s triad. Physical examination showed a temperature of 40 °C, heart rate of 120/min, blood pressure of 14/9 cmHg, icterus and right upper quadrant tenderness. On blood tests, leukocyte count was 22,770/ml, C-reactive protein level260 mg/l, and conserved renal function. His liver function tests revealed a total bilirubin of 355 umol/l, conjugated bilirubin of 195 umol/l, an ALT of 215 U/l, a γ-GT of 729 U/l and an alkaline phosphatase of 126 U/l. Prothrombin time was 50%. On ultrasonography, we noted dilatation of both the extrahepatic and intrahepatic biliary tract. CT scan revealed a hyper-dense obstacle of the lower bile duct (Fig. 3).

**Fig. 4:**
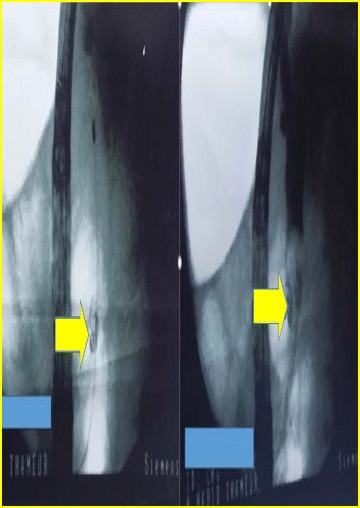
ERCP, before (*Left picture*) and after (*Right picture*) contrast agent injection, showing the coil (*Yellow arrow*) in the lower main bile duct

ERCP showed the presence, in the lower bile duct, of an intraluminal coil and stones, with upstream biliary tree dilatation (Fig. 4). An extraction of the coil and fragmented stones was carried out and vacuity of bile ducts was attained. Following this, the patient recovered uneventfully and was discharged 8 days later.

## Discussion

### Etiology of GDA pseudo-aneurysms

Aneurysms of GDA are very rare. They are estimated to be less than 1.5% of all splanchnic artery aneurysms [2]. They are divided into two types: True aneurysms and pseudo-aneurysms like our case. GDA pseudo-aneurysms are generally secondary to acute or chronic pancreatitis, cholangitis, traumatic or iatrogenic causes [3–6]. All these factors were present in our case. It is most likely that the iatrogenic factor in an inflammatory environment precipitated the formation of the pseudo-aneurysm. This is the second case of GDA pseudo-aneurysm after choledochotomy in the English literature. The first case was reported byEkeland and al in 1974 [7].

### Symptomatology

GDA pseudo-aneurysms could remain silent for a long time and be revealed accidentally in an imaging study. On the contrary, theycould be symptomatic. Revelation modes are mainly compression, and bleeding. Bleeding is considered to be the most serious complication, as it can rapidly lead to a hemorrhagic shock, and ultimately death. It can occur after the rupture of the pseudo-aneurysm in the peritoneum, the retroperitoneum or in the gastrointestinal tract by way of the duodenum or the bile duct causing haemobilia like in our case [8].

Rupture of a GDA pseudo-aneurysm in the main bile duct is exceedingly rare. Only few cases in the literature have been reported [4]. Symptoms of haemobilia are, classically, abdominal pain, upper gastrointestinal hemorrhage and jaundice. The complete triad known as Quincke’s triad occurred only in 22% of 222 cases of haemobilia, in the review of Green and al. [9]. Our patient presented this triad, which adds to the particularities of this case.

### Diagnosis and treatment

In our case, the patient presented to our institution, 2 weeks after biliary surgery, with upper gastrointestinal bleeding. Usedexplorations to detect an etiology for this bleeding, including upper gastrointestinal endoscopy and lateral duodenoscopy, were inconclusive. This is may be due to the fact that the patient stopped bleeding as would suggest the hemodynamic and hemoglobin level stabilities. Therefore, the intermittence of a bleeding could be a source of false negatives if sensitive diagnostic tools are not used. In the recent literature, only 12% of haemobilia cases were diagnosed endoscopically [10].

Arteriography is the most valuable diagnostic test to detect Splanchnic artery aneurysms and the exact location of bleeding [11, 12]. However, this technique requires a trained interventional radiologist. CT angiography and magnetic resonance angiography could be also used in the diagnostic arsenal, when arteriography is not available, with high threshold of detection of Splanchnic artery aneurysms [13].

Management of GDA pseudo-aneurysms relies either on surgery or angiographic embolization. The latter, if available, is considered the safer and more effective option, especially in the active bleeding patient [13, 14]. Success rate is estimated to be around 80 to 100% [9]. A variety of embolic agents have been used. In our case, we used three metallic coils with preserved permeability of the gastroduodenal artery. Nevertheless,surgery remains the only option for proximal GDA pseudo-aneurysms [4] and should be considered if angiographic embolization fails or is unavailable or contraindicated [15, 16].

### Rare complication after GDA embolization

After embolization of Splanchnic artery aneurysms, some complications could occur. In the short term, accidentalembolization of the wrong vessel with ensuing infarction is may be the most serious complication. In the long term, complications are rare. In our case, 20 months after embolization, the patient presentedwith cholangitis secondary to coil migration from the embolized GDA pseudo-aneurysm. To our knowledge, this is the first case to be published in the English literature. Some cases of cholangitis after coil embolization of hepatic arteries were reported [17–19]. The best management of these conditions is through ERCP, especially in severe cholangitis like in our case. AS for surgery, it could be indicated after ERCP failure.

## Conclusion

GDA pseudo-aneurysms are very rare. Bleeding, secondary to the rupture of these lesions, is a serious complication that could lead to death. Thus, surgeons must keep a high index of suspicion, in the set of patients with history of biliary surgery, presenting with upper gastrointestinal bleeding. Diagnosis and treatment of ruptured GDA pseudo-aneurysms, rely on angiography. This method is considered to be safe. Cholangitis secondary to coil migration in the main bile duct is exceedingly rare,but remains an eventuality that physicians should be cognizant of.

## Abbreviations

ALP: Alkaline phosphatase
ALT: Alanine transaminase
ERCP: Endoscopic retrograde cholangiopancreatography
GDA: Gastroduodenal artery
γ-GT: Gamma-glutamyltranspeptidase
